# CHLA 2026 CONFERENCE INTERACTIVE WORKSHOPS / ABSC CONGRÈS 2026 ATELIERS INTERACTIFS

**DOI:** 10.29173/jchla29970

**Published:** 2026-08-03

Michelle Price

St. John Fisher University

**Topic:** Library patrons do not enter one-on-one consultations as fragmented people. They bring their whole selves to the interaction, including their trauma. How does a librarian help a patron, staying within our scope of practice, but also accept and address the trauma that could be constraining the interaction. This workshop will focus on synchronous patron interactions, held physically or virtually. The Substance Abuse and Mental Health Services Administration (SAMHSA) has developed the SAMHSA's Concept of Trauma and Guidance for a Trauma-Informed Approach that can serve as a guide for reframing service in this setting. The approach includes the three Es of trauma, the four Rs that are key assumptions, the six key principles, and the ten implementation domains for systems. This adoption is a no-tech solution that only requires practitioner reflection and intent for implementation. **Target Audience**: Librarians at any career stage, interacting individually with patrons in all types of settings and modalities. **Objectives:** 1. Identify the SAMSHA Concept of Trauma Guidance for a Trauma-Informed Approach, including the concept of trauma, key assumptions, and the guiding principles for approaching trauma, 2. Assess current personal practice against the SAMSHA Approach, 3. Construct trauma-informed practices for synchronous, one-on-one interactions with patrons. **Interactive Activities:** Scripted role-playing, critical incident method, and autoethnographic reflection will be used during the session.

