# Supplementary material for: CHLA 2026 CONFERENCE INTERACTIVE WORKSHOPS / ABSC CONGRÈS 2026 ATELIERS INTERACTIFS
Source: J Can Health Libr Assoc. 2026 Aug 3;47(2):77–8. doi: 10.29173/jchla29970 (PMC13590148; doi:10.29173/jchla29970)
Supplement: Related texts

# Abstract: IW2. Making the Library Value Planner your ultimate strategic planning MVP

Mark Mueller^1^, Joanne Hodder^2^, Jeanna Hough^3^, Vinson Li^2^, Iveta Lewis^4^

^1^Saskatchewan Health Authority, ^2^Nova Scotia Health, ^3^Halton Health Ontario, ^4^Holland Bloorview Kids Rehabilitation Hospital Ontario

**Topic:** The CHLA/ABSC Library Value Planner (LVP): Your Ultimate Strategic Planning Tool - this Tool has been developed by the CHLA Standing Standards Committee. **Target Audience:** Health Library professionals including Managers, leaders & other Library professionals. **Objectives:** This interactive workshop aims to equip library professionals with the skills to create and communicate a strategic plan that demonstrates the value of their libraries and their roles. Participants will learn to use the LVP to navigate challenges and effectively advocate for their library's role in health care settings. This tool will help librarians be recognized for their role in driving innovation and collaboration, while incorporating principles of DEIA to support evidence-based healthcare practices. **Interactive activities:** 1. Strategic planning initial simulation, 2. LVP: employ & complete, 3. Applying actionable communication tools.

# Abstract: IW3. Understanding AI agents and their role in systematic reviews

Ghayath Janoudi, Yuhong Yuan, Mara Rada Loon

**Topic:** AI agents and agentic AI systems represent a new architectural paradigm for applying Generative AI in workflows that require reasoning, information retrieval, and decision support. Unlike earlier forms of GenAI that focused primarily on text generation, agentic systems integrate retrieval, ranking, planning, and tool-use to deliver more reliable, verifiable, and domain-specific outputs. Their potential impact on information access and evidence synthesis is rapidly expanding. **Activity Description:** In this 90-minute workshop, participants will first develop a foundational understanding of key terminology, core concepts, and the current landscape of agentic AI. We will then explore real-world examples demonstrating how agentic AI systems are being used in systematic reviews—including eligibility criteria development, search strategy, citation screening, data extraction, and validation workflows. We will focus on the application of search strategy creation and showcase how the audience can build a search strategy with the assistance of AI agents. Finally, we will examine practical frameworks for critically appraising these systems, focusing on validity, performance claims, biases, and evaluation methodologies. **Objectives:** Participants will leave with: A clearer understanding of what AI agents and agentic systems are (and what they are not), realistic expectations about their potential value and limitations in health libraries and evidence synthesis, practical skills to evaluate claims, ask informed questions, and support decision-making in their institutions. **Target Audience:** This session is designed for health librarians, information specialists, and anyone interested in the future of AI-supported evidence synthesis.

# Abstract: IW4. Search strategies as data: navigating the realities of control, deposit, attribution, and advocacy in knowledge synthesis

Sabine Calleja^1^, Jill Boruff^1^, Heather Cunningham^2^, Karly Gunson^3^, Julia Martyniuk^2^, Ani Orchanian-Cheff^4^, Alix Pincivy^5^, Alisa B. Rod^1^, Daniela Ziegler^6^

^1^McGill University, ^2^University of Toronto, ^3^University of Saskatchewan, ^4^University of Toronto, University Health Network, ^5^CHU Sainte-Justine, ^6^Centre hospitalier de l'Université de Montréal

**Topic:** This workshop builds on ongoing discussions around understanding knowledge synthesis (KS) search strategies as data and promoting their deposit in research data repositories. It will foster dialogue and critical thinking through real-world scenarios that health sciences librarians frequently encounter when searching for, developing, citing, and advocating for librarian-authored search strategies in KS projects. Through group discussions, scenario-based activities, and collective brainstorming, attendees will deepen their understanding of: The value of librarian-authored search strategies as intellectual outputs; the importance of depositing librarian KS work in research data repositories; common barriers to deposit and authorship recognition; practical strategies for advocacy and institutional change. **Target Audience:** Health sciences librarians in any setting who develop search strategies for KS projects, have questions related to authorship, recognition, or data deposit and/or are curious about how to advocate for the visibility and impact of their KS-related work. **Objectives:** By the end of this workshop, participants will be able to articulate the benefits and challenges of conceptualizing search strategies as research data; identify common barriers to depositing search strategies and receiving appropriate credit; analyze real-world scenarios to develop practical responses and advocacy strategies; collaborate with peers to brainstorm solutions and share institutional practices. **Activity Description:** In small groups participants will engage in mini design-thinking activities to discuss real-world scenarios, identify key issues, and propose possible solutions.
